# Sarcopenic Obesity and Sex-Specific Functional Impairment in Older Adults: An Observational Case–Control Study

**DOI:** 10.3390/jcm15176866

**Published:** 2026-09-04

**Authors:** Stefano Lazzer, Lara Mari, Mattia D’Alleva, Jacopo Stafuzza, Simone Zaccaron, Nicola Campigotto, Enrico Rejc, Gabriella Tringali, Ilaria Grimoldi, Roberta De Micheli, Adele Bondesan, Ferruccio Nibbio, Paolo Fanari, Alessandro Sartorio

**Affiliations:** 1 Department of Medicine, University of Udine, 33100 Udine, Italy; mari.lara@spes.uniud.it (L.M.); mattia.dalleva@uniud.it (M.D.); jacopo.stafuzza@uniud.it (J.S.); simone.zaccaron@uniud.it (S.Z.); nicola.campigotto@uniud.it (N.C.); enrico.rejc@uniud.it (E.R.); 2 School of Sport Sciences, University of Udine, 33100 Udine, Italy; 3 Department of Theoretical and Applied Sciences, eCampus University, Novedrate, 22060 Como, Italy; 4 Department of Neurosciences, Biomedicine and Movement Sciences, University of Verona, 37129 Verona, Italy; 5 Experimental Laboratory for Auxo-Endocrinological Research, Istituto Auxologico Italiano, IRCCS (Istituto di Ricovero e Cura a Carattere Scientifico), Piancavallo, 28824 Verbania, Italy; g.tringali@auxologico.it (G.T.); i.grimoldi@auxologico.it (I.G.); r.demicheli@auxologico.it (R.D.M.); a.bondesan@auxologico.it (A.B.); sartorio@auxologico.it (A.S.); 6 Division of Cardiological Rehabilitation, Istituto Auxologico Italiano, IRCCS (Istituto di Ricovero e Cura a Carattere Scientifico), Piancavallo, 28824 Verbania, Italy; f.nibbio@auxologico.it; 7 Division of Pneumological Rehabilitation, Istituto Auxologico Italiano, IRCCS (Istituto di Ricovero e Cura a Carattere Scientifico), Piancavallo, 28824 Verbania, Italy; p.fanari@auxologico.it

**Keywords:** ageing, sarcopenic obesity, sex differences, visceral adipose tissue, functional capacity, body composition, quality of life

## Abstract

**Background**: Sarcopenic obesity (SO) is a clinically relevant but often under-recognized condition in older adults. This study examined whether obesity severity and body fat distribution are associated with physical capacity, functional autonomy and health-related quality of life in a sex-specific manner. **Methods**: In this observational case–control study with cross-sectional assessments, 45 older obese adults (OB; 27 men and 18 women) and 40 non-obese (NOB) comparison participants (18 men and 22 women), aged 65–85 years, underwent anthropometric assessment, bioelectrical impedance analysis, geometric body shape indexing, physical performance testing, autonomy assessment, physical activity evaluation and health-related quality-of-life assessment. SO was classified using a study-specific operational application of the ESPEN/EASO framework based on excess adiposity, impaired muscle function, and BIA-derived skeletal muscle mass adjusted for body mass. **Results**: Using this operational framework, all participants in the OB group were classified as presenting an SO phenotype. OB men showed substantially greater visceral adipose tissue mass than OB women (+106.5%, ES: 1.27, *large*, *p* < 0.001) and a higher A body shape index-associated relative risk (+27.9%, ES: 0.82, *large*, *p* < 0.001). Conversely, OB women showed more severe mobility impairment than OB men, with lower performance in the 8-Foot Up-and-Go (−22.9%, ES: 0.66, *medium*, *p* < 0.001) and 6-Min Walk test (−23.4%, ES: 0.64, *medium*, *p* < 0.001) and slightly lower basic autonomy. Health-related quality of life was significantly reduced only in OB men (−10.8%, ES: 1.07, *large*, *p* < 0.001) than NOB. **Conclusions**: In this clinical sample, an operational SO phenotype was identified among older OB adults and exhibited divergent sex-specific profiles. Men showed a predominantly geometric visceral risk phenotype and poorer perceived health status, whereas women showed more pronounced impairment in mobility and basic functional autonomy. These findings support sex-differentiated, multidisciplinary rehabilitation strategies in older adults with obesity.

## 1. Introduction

Population ageing and the increasing prevalence of obesity are converging to create a major clinical and public health burden [1,2]. In older adults, obesity is not only a disorder of excess body mass; it is also associated with metabolic dysfunction, chronic low-grade inflammation, reduced mobility, osteoarthritis, cardiometabolic disease and a higher risk of disability [3,4,5]. These effects are amplified when obesity coexists with impaired muscle function and reduced muscle mass, a phenotype now defined as sarcopenic obesity (SO) [6,7].

SO is particularly relevant in geriatric and rehabilitation settings because excess adiposity and muscle impairment reinforce each other. Low physical activity reduces mechanical loading and accelerates loss of muscle strength. At the same time, excess fat mass increases the energy cost of movement and contributes to ectopic fat accumulation in and around skeletal muscle [8]. This creates a self-perpetuating cycle of fatigue, reduced activity, muscle quality deterioration and progressive loss of independence.

Body mass index (BMI) remains widely used for clinical screening, but it has important limitations in older adults. BMI cannot distinguish fat mass (FM) from fat-free mass (FFM) and does not characterize abdominal or visceral adiposity, both of which become increasingly relevant with ageing [9]. For this reason, complementary indices such as waist-to-height ratio (WHtR) [10], body roundness index (BRI) [11], estimated visceral adipose tissue (VAT) mass, and A body shape index (ABSI) may improve phenotyping of adiposity-related risk [12,13]. These indices may be particularly useful when evaluating sex-specific phenotypes, because older men and women differ in fat distribution, muscle reserves and functional thresholds [14].

The clinical consequences of SO are best captured by combining body composition measures with objective functional tests and validated autonomy scales. The senior fitness test battery [15], handgrip strength, the physical performance test (PPT) [16], basic (BADL) and instrumental (IADL) activities of daily living [17,18], the Barthel index (BI) [19], the physical activity scale for the elderly (PASE) [20] and the 12-item short-form health survey (SF-12) provide complementary information on physical capacity, daily autonomy, lifestyle activity and perceived health [21].

Recent evidence has sharpened both the clinical relevance and the methodological challenges of SO [22]. The ESPEN/EASO consensus recommends a sequential diagnostic approach in which low skeletal muscle function is followed by body composition confirmation of excess adiposity and reduced skeletal muscle mass relative to body size. Contemporary reviews emphasize that prevalence estimates and prognostic associations remain highly sensitive to the selected muscle function and body composition criteria [23]. In an obesity cohort, for example, estimated SO prevalence varied markedly according to whether fixed handgrip thresholds, age- and sex-adjusted handgrip scores, or chair stand performance were used [24]. Despite growing evidence linking SO to impaired physical performance and adverse geriatric and cardiometabolic outcomes, comparatively few studies have examined whether visceral or geometric adiposity, mobility, autonomy, physical activity, and perceived health cluster differently in older men and women. This unresolved issue provides the rationale for the present sex-stratified, multidomain analysis.

The primary objective of this study was to evaluate how obesity severity and phenotypic fat distribution, characterized by anthropometric and geometric indices, relate to physical capacity, functional independence and health-related quality of life in older adults. A secondary objective was to determine whether these associations reveal distinct sex-specific trajectories. We hypothesized that, among older obese adults, men would show a greater visceral and geometric risk profile, whereas women would show more pronounced impairment in mobility and autonomy.

## 2. Materials and Methods

### 2.1. Study Design and Participants

This observational case–control study included 45 older obese adults (OB; 27 men and 18 women) recruited before admission to a 3-week inpatient, multidisciplinary weight management program at the Istituto Auxologico Italiano, Istituto di Ricovero e Cura a Carattere Scientifico (IRCCS), Verbania-Piancavallo, Italy, a third-level center for care and rehabilitation of obesity and nutritional disorders. A comparison group of 40 non-obese (NOB) older adults (18 men and 22 women) was recruited by the School of Sport Sciences, at the University of Udine (Udine, Italy) from local cultural associations and were not selected according to physical activity level, fitness status, or participation in structured exercise programs. Eligible participants were aged 65–85 years. The exclusion criterion was the inability to complete the functional assessments safely. The two groups were recruited independently and were not individually matched for age, sex, physical activity level, or fitness status. No additional clinical eligibility criteria were applied for the present analysis beyond the stated age range and the ability to safely complete the study assessments.

The study protocol was approved by the Territorial Ethical Committee 5, Lombardy Region, Milan, Italy (protocol number: 144/25; date of approval: 1 April 2025; research project code: 01C514). The study was conducted in accordance with the Declaration of Helsinki. All participants provided written informed consent before enrolment.

### 2.2. Study Protocol

Testing was conducted between April 2025 and March 2026. OB participants were assessed immediately before starting the inpatient program. NOB participants completed all assessments during a single laboratory visit at the University of Udine. The same trained operators performed all assessments to minimize inter-examiner variability. The protocol included anthropometry, body composition, geometric adiposity indices, physical capacity tests, functional autonomy scales, physical activity assessment and health-related quality-of-life assessment.

### 2.3. Anthropometry and Body Shape Indices

Body mass (BM) was measured to the nearest 0.1 kg using a calibrated electronic scale (SECA, Hamburg, Germany), and standing height (Ht) was measured with a Harpenden stadiometer (Holtain Limited, Crymych, Dyfed, UK). Body mass index (BMI) was calculated as body mass divided by height squared (kg/m^2^). Waist circumference (WC) was measured midway between the lowest rib and the iliac crest using a non-elastic tape according to World Health Organization procedures [25]. 

Abdominal adiposity was evaluated using the waist-to-height ratio (WHtR), a validated screening tool for central obesity and related cardiometabolic risks [10], calculated as

$$\mathrm{WHtR}=\frac{Waist\;circumference\;(cm)}{Height\;(cm)}$$

A threshold value of 0.50 was used to identify elevated visceral fat accumulation and cardiovascular risk.

To integrate global adiposity with regional fat distribution, the body mass fat index (BMFI) was calculated using the following equation [26]:

BMFI = BMI (kg/m^2^) × FM (%) × WC (cm)

where FM denotes total body fat percentage estimated by bioelectrical impedance analysis, as described in Section 2.4.

The body roundness index (BRI) was determined to assess body shape eccentricity, independent of traditional anthropometric boundaries, using the geometric equation proposed by Thomas et al. [11]:

$$\mathrm{BRI}\;=\;364.2\;-\;365.5\;\times \;\sqrt[{2}]{1-({\frac{WC}{2\pi \;\times \;0.5\;\times \;Height})}^{2}}$$

where WC and height are expressed in meters. Higher BRI scores reflect a rounder body shape associated with increased visceral adiposity.

Visceral adipose tissue (VAT) mass was estimated using the predictive geometrical framework [11]. Body eccentricity (e), modelling the human body shape as an ellipse, was calculated as follows:

$$e\;=\;\sqrt[{2}]{1-({\frac{WC}{2\pi \;\times \;0.5\;\times \;Height})}^{2}}$$

The eccentricity value was incorporated into multivariable regression models to predict VAT volume (L) [11]. Volume was subsequently converted into absolute VAT mass (kg) by applying the validated tissue density constant [27]:

VAT Mass (kg) = VAT Volume (L) × 1.09 (kg/L)

Relative visceral adiposity was expressed as a percentage of total fat mass:

$$\mathrm{VAT}\;(\%)\;=\;\frac{VAT\;mass\;(kg)}{FAT\;mass\;(kg)}\;\times \;100$$

To quantify geometric-associated mortality hazards, the A body shape index relative risk (ABSI-RR) was initially computed from WC (m), height (m), and BMI (kg/m^2^) [12]:

$$\mathrm{ABSI}\;=\;\frac{WC}{{BMI}^{2/3}\;\times \;{Height}^{1/2}\;}$$

The ABSI z-score was calculated by standardizing individual values against age- and sex-specific population averages (μ_ABSI_ and σ_ABSI_) derived from reference data [12]:

$$\mathrm{ABSI}\;z\text{-}\mathrm{score}\;=\;\frac{ABSI\;-\;\mu ABSI\;}{\sigma ABSI\;}$$

Finally, the relative risk was estimated using the proportional hazards model:

$$\mathrm{ABSI}\text{-}\mathrm{RR}\;=\;e^{\beta \;\times \;ABSI\;z-score}$$

where β ≈ 0.33 represents the log hazard ratio per standard deviation increase in ABSI. An ABSI-RR > 1 indicates an elevated mortality risk relative to the population mean.

### 2.4. Body Composition

Body composition was assessed by bioelectrical impedance analysis (Human-IM Scan, DS-Medigroup, Milan, Italy) following standardized procedures [28]. Participants rested supine for 20 min before measurement, with limbs slightly abducted. Absolute and relative fat-free mass (FFM) and fat mass (FM) were recorded. To minimize variability related to hydration, all measurements were obtained in the morning following an overnight fast and bladder emptying. Participants were assessed only in the absence of clinical evidence of peripheral edema or acute electrolyte disturbances. Fat-free mass (FFM) and fat mass (FM) were estimated in all participants using the manufacturer’s validated specific prediction equations.

### 2.5. Physical Capacity and Functional Autonomy

Handgrip strength (HGS) was measured with a calibrated hydraulic Jamar dynamometer (Lafayette Instrument Company, Lafayette, IN, USA). Participants performed three maximal contractions with each hand, separated by 60 s of rest. The highest value obtained from either hand was used for the operational muscle function classification [29].

Functional fitness was evaluated using the senior fitness test battery [15], including the 30 s chair stand, arm curl, 2 min step, chair sit-and-reach, back scratch, 8-foot up-and-go and 6-Min Walk test. 

Global physical performance was assessed using the 7-item Physical Performance Test, which evaluates fine- and gross-motor tasks relevant to daily living [18].

Functional independence was assessed using the following: Basic activities of daily living (BADL), which evaluates independence across six domains (bathing, dressing, toileting, transferring, continence, feeding) scored from 0 to 6 [17]. Instrumental activities of daily living (IADL), which assesses complex community living skills across eight domains (telephone use, shopping, food preparation, housekeeping, laundry, mode of transportation, medication responsibility, and financial management) scored from 0 to 8 [18]. Barthel index (BI), which measures the degree of independence and assistance required across ten self-care and mobility items (feeding, bathing, grooming, dressing, bowel continence, bladder continence, toilet use, transfers bed to chair, level-ground mobility, and stair climbing), yielding a total score from 0 to 100 [19].

### 2.6. Physical Activity and Health-Related Quality of Life

Self-reported physical activity was assessed with the physical activity scale for the Elderly (PASE), a 12-item questionnaire that captures leisure, household and occupational/voluntary activities during the previous 7 days [20]. Health-related quality of life was assessed with the 12-item short-form health survey (SF-12), which provides a standardized summary of perceived physical and mental health [21].

### 2.7. Sarcopenic Obesity Classification

SO was classified using a study-specific operational application of the ESPEN/EASO framework [6]. For screening, excess adiposity was identified by BMI ≥ 30 kg/m^2^; WC was also recorded as an indicator of central adiposity. Diagnostic confirmation required the concurrent presence of impaired skeletal muscle function and altered body composition. Impaired muscle function was defined as handgrip strength <27 kg in men and <16 kg in women, or a 30 s chair stand performance at or below the age- and sex-specific reference threshold (men: ≤16, ≤15, ≤14, ≤13, and ≤11 repetitions for ages 65–69, 70–74, 75–79, 80–84, and 85–89 years, respectively; women: ≤15, ≤14, ≤13, ≤12, and ≤11 repetitions, respectively). Altered body composition was defined by excess BIA-derived fat mass (>30% in men and >40% in women) together with reduced skeletal muscle mass relative to body mass (SMM/BM < 37.0% in men and <27.6% in women) [6]. Participants meeting these operational criteria were classified as presenting an SO phenotype. Because body composition was assessed by BIA and the available study measures represent an operational implementation of the consensus algorithm, this classification was not interpreted as definitive clinical confirmation of SO according to the complete ESPEN/EASO diagnostic pathway.

### 2.8. Statistical Analysis

Statistical analyses were performed using R software (version 4.5.2; R Foundation for Statistical Computing, Vienna, Austria) and GraphPad Prism 10.0.1. Normality was assessed with the Shapiro–Wilk test. Assumptions of linearity, homoscedasticity, and normality of residuals were verified using Shapiro–Wilk tests. When assumptions were violated, Welch’s *t*-test or non-parametric sensitivity analyses were considered. 

Anthropometric, body composition, and functional variables were analyzed using a two-way analysis of variance with Group (OB vs. NOB), Sex (men vs. women) and Group × Sex interaction as fixed factors. Post hoc pairwise comparisons were adjusted with Tukey’s honest significant difference test. The magnitude of between-group differences was interpreted using conventional Cohen’s d thresholds: *small* effect (d = 0.20–0.49), *medium* effect (d = 0.50–0.79), and *large* effect (d ≥ 0.80) [30,31].

**Figure 1:**
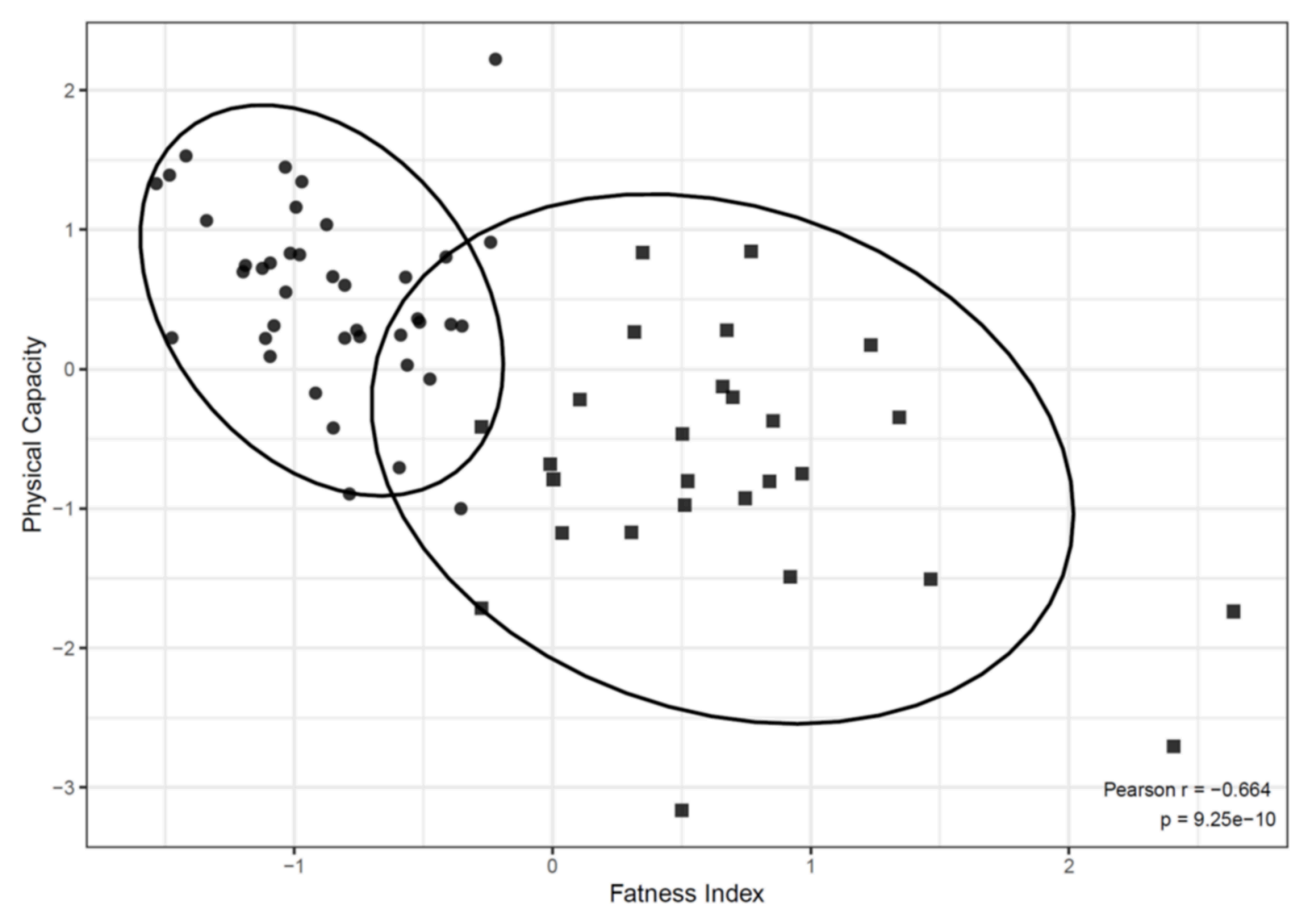
Association between composite physical capacity score (hand grip, senior fitness test) and composite fatness score (waist-to-height ratio; body mass fat index; body roundness index; visceral adipose tissue; a body shape index) across obese subjects (OB, ■) and non-obese controls (NOB, ●). r = −0.66, *p* < 0.001, with 95% confidence ellipses shown.

**Figure 2:**
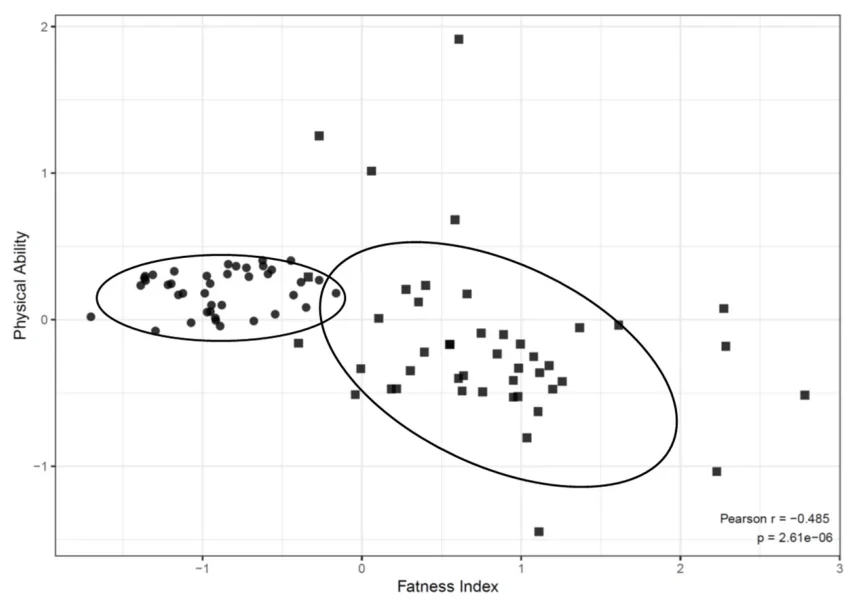
Association between composite physical ability score (PPT, BADL, IADL, BI) and composite Fatness score (waist-to-height ratio; body mass fat index; body roundness index; visceral adipose tissue; a body shape index) across obese subjects (OB, ■) and non-obese controls (NOB, ●). r = −0.49, *p* < 0.001, with 95% confidence ellipses shown.

**Figure 3:**
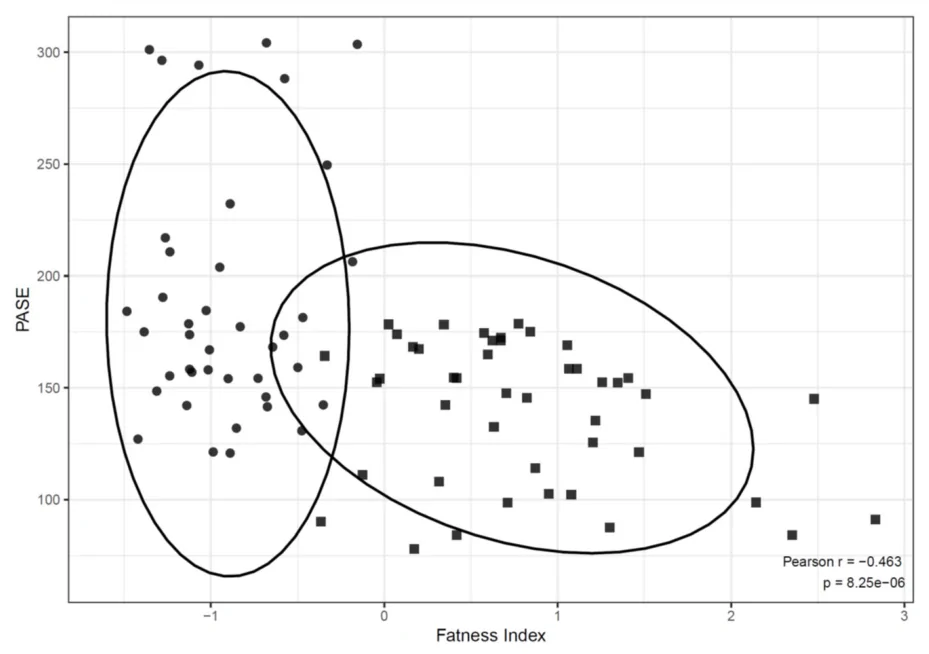
Association between physical activity scale for the elderly (PASE) and composite fatness score (waist-to-height ratio; body mass fat index; body roundness index; visceral adipose tissue; a body shape index) across obese subjects (OB, ■) and non-obese controls (NOB, ●). r = −0.46, *p* < 0.001, with 95% confidence ellipses shown.

**Figure 4:**
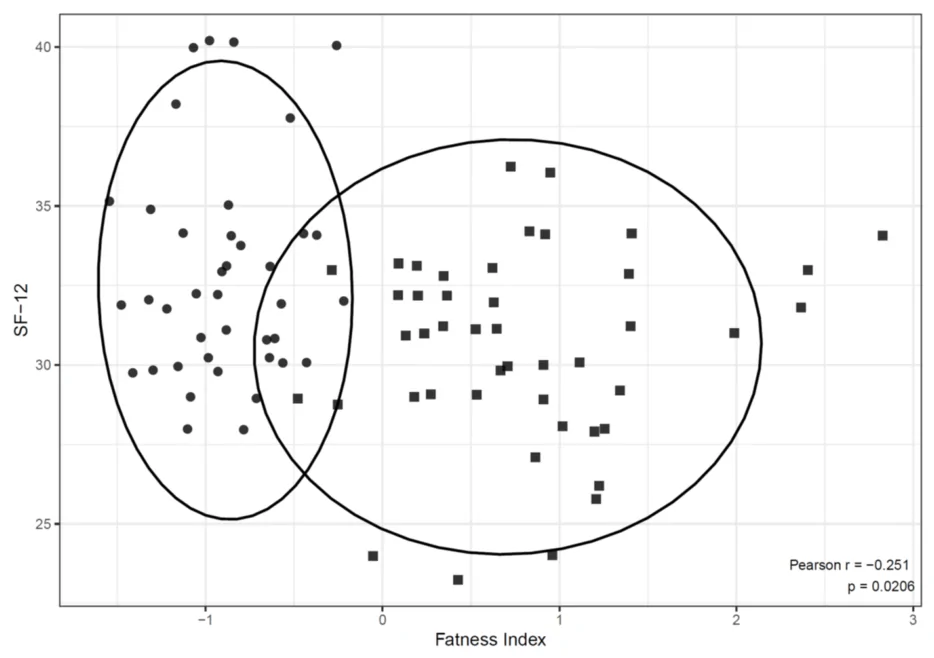
Association between 12-item short-form health survey (SF-12) and composite fatness score (waist-to-height ratio; body mass fat index; body roundness index; visceral adipose tissue; a body shape index) across obese subjects (OB, ■) and non-obese controls (NOB, ●). r = −0.25, *p* = 0.020, with 95% confidence ellipses shown.

To reduce data dimensionality and obtain synthetic indices representative of each domain, principal component analysis (PCA) was performed after z-score standardization for fatness, physical capacity, and physical ability. The fatness PCA included waist-to-height ratio, body mass fat index, body roundness index, visceral adipose tissue mass, and A Body Shape Index. The physical capacity PCA included right and left handgrip strength and the seven variables of the senior fitness test. The physical ability PCA included PPT, BADL, IADL, and BI. For the composite indices used in the graphical analyses, each participant’s score corresponded to the first principal component (PC1), calculated as the weighted linear combination of the standardized input variables using the PCA coefficients. Component orientation was chosen so that higher fatness scores represented greater adiposity, whereas higher physical capacity and physical ability scores represented better function. PASE and SF-12 were single questionnaire-derived variables and were therefore not subjected to PCA; their original scores were used in Figures 3 and 4, respectively. The adequacy of the dataset for PCA was evaluated using the Kaiser–Meyer–Olkin measure and Bartlett’s test of sphericity. Components were retained based on eigenvalues > 1, scree plot inspection, and the proportion of explained variance. For each retained component, eigenvalues, percentage and cumulative explained variance, component loadings, and communalities were reported. Statistical significance was set at *p* < 0.05. Figure 1, Figure 2, Figure 3 and Figure 4 were intended to provide a descriptive graphical overview of the overall relationships between adiposity and functional outcomes across the study population, whereas detailed group- and sex-specific comparisons are reported in Table 1 and Table 2. Complete data were available for all 85 participants for the variables reported in Table 1 and Table 2 and included in the PCA analyses; therefore, no missing data imputation or case-wise exclusion was required.

## 3. Results

### 3.1. Anthropometry and Body Composition

The anthropometric and body composition profiles of the subjects are summarized in Table 1. Using the study-specific operational application of the ESPEN/EASO framework, all participants in the OB group were classified as presenting an SO phenotype; mean SMM/BM was 17.1 ± 3.1% in men and 12.0 ± 1.3% in women (*p* < 0.001). No significant age differences were found between OB and NOB men, whereas OB women were significantly older (+8.6%, ES: 1.80, *large*, *p*: 0.032) than NOB women (Table 1). No significant difference was observed in the OB group, while NOB men were significantly older (+5.0%, ES: 0.99, *large*, *p*: 0.021) than NOB women.

BM was significantly higher in both OB men (+60.6% vs. NOB men, ES: 3.00, *large*, *p* < 0.001) and OB women (+55.1% vs. NOB women, ES: 2.7, *large*, *p* < 0.001). Additionally, BM in men was significantly higher than in women in both the OB (+20.6%, ES: 1.13, *large*, *p* < 0.001) and NOB (+16.5%, ES: 1.27, *large*, *p* < 0.001) groups. 

Similarly, BMI was significantly higher in both OB men (+67.1% vs. NOB, ES: 3.84, *large*, *p* < 0.001) and OB women (+74.2% vs. NOB, ES: 4.11, *large*, *p* < 0.001), but it did not differ significantly between sex within either the OB and NOB groups. 

Central adiposity index was strongly driven by the group effect (Table 1). WC was significantly higher in OB men (+49.4%, ES: 4.15, *large*, *p* < 0.001) and OB women (+39.0%, ES: 4.40, *large*, *p* < 0.001) relative to their respective NOB peers. Regarding sex, OB men exhibited a significantly higher WC (+11.4%, ES: 1.16, *large*, *p*: 0.038) than OB women, whereas WC did not differ between groups in NOB subjects. 

The WHtR was significantly higher in OB men (+51.0%, ES: 4.64, *large*, *p* < 0.001) and OB women (+48.0%, ES: 4.21, *large*, *p* < 0.001) compared to NOB, with no sex differences recorded in either group.

Global adiposity indices, specifically the body mass fat index and body roundness index (Table 1), showed higher values in both OB men (+375.0% and +191.9%; ES: 3.09, *large* and ES: 3.73, *large*; respectively, *p* < 0.001) and OB women (+323.7% and +166.2%; ES: 3.40, *large* and ES: 3.95, *large*; respectively, *p* < 0.001) compared to NOB subjects. Neither index showed significant sex differences within the OB or NOB groups. 

Absolute VAT mass (Table 1) was significantly higher in OB men (+1441.0%, ES: 2.40, *large*, *p* < 0.001) and OB women (+401.7%, ES: 3.17, *large*, *p* < 0.001), relative to the NOB subjects; concurrently, higher relative VAT% (+300.0% in OB men, ES: 3.05, *large*; +90.3% in OB women, ES: 2.36, *large*; vs. NOB peers, *p* < 0.0011). Additionally, OB men had a 106.5% (ES: 1.27, *large*) higher absolute VAT mass and an 89.8% (ES: 1.99, *large*) higher relative VAT% compared to OB women (*p* < 0.0011), while no significant sex differences were observed among NOB subjects.

Furthermore, ABSI was significantly higher in OB men (+12.5% vs. NOB men, ES: 1.00, *large*, *p*: 0.018) but did not differ significantly between OB women and NOB women (Table 1). Consequently, in the sex comparison, OB men showed a higher ABSI (+12.5%, ES: 1.00, *large*, *p*: 0.036) and a higher ABSI-associated relative risk (+27.9%, ES: 0.82, *large*, *p* < 0.001) compared to OB women. In contrast, no sex differences emerged within the NOB group.

Absolute FFM was significantly higher in OB men (+16.7% vs. NOB men, ES: 1.12, *large*, *p*: 0.022) but not in OB women; however, the relative FFM% was significantly lower in both OB men (−26.3%, ES: 3.68, *large*, *p* < 0.001) and OB women (−30.9%, ES: 3.79, *large*, *p* < 0.001) compared to NOB subjects. In terms of sex differences, OB men showed higher values of FFM (+43.8%, ES: 3.16, *large*, *p* < 0.001) and relative FFM% (+20.4%, ES: 1.60, *large*, *p* < 0.001) than OB women. NOB men maintained a significantly higher absolute FFM (+30.0%, ES: 1.52, *large*, *p* < 0.001) and relative FFM% (+12.8%, ES: 1.74, *large*, *p*: 0.019) than NOB women.

Absolute FM (kg) (Table 1) was significantly higher in both OB men (+206.9% vs. NOB, ES: 3.40, *large*, *p* < 0.001) and OB women (+171.0% vs. NOB, ES: 3.23, *large*, *p* < 0.001), with no absolute FM differences between sex in either cohort. Conversely, relative FM% was significantly higher in both OB men (+91.5% vs. NOB, ES: 3.79, large, *p* < 0.001) and OB women (+68.8% vs. NOB, ES: 3.81, large, *p* < 0.001), with women physiologically exhibiting higher relative adiposity than men in both the OB (+23.0%, ES: 1.62, *large*, *p* < 0.001) and NOB (+39.5%, ES: 1.80, *large*, *p* < 0.001) groups.

Overall, group status explained most variation in global and central adiposity, whereas sex differences within the OB group were concentrated in visceral adiposity and ABSI-related risk, which were higher in men; women had higher relative fat mass.

### 3.2. Physical Capacities

Muscular strength and functional performance were impaired in the OB group (Table 2). Handgrip strength was significantly lower in both OB men (−27.6%, ES: 0.96, *large*, *p* < 0.001) and OB women (−55.3%, ES: 1.91, *large*, *p* < 0.001) compared to their NOB counterparts. A comparable functional decline was observed in lower body strength (30-s chair stand), with a significant lower repetition in OB men (−31.2%, ES: 1.21, *large*, *p* < 0.001) and OB women (−46.3%, ES: 2.57, *large*, *p* < 0.001) vs. NOB subjects; as well as in upper body function (arm curl), which decreased by 21.5% (ES: 0.90, *large*, *p* < 0.001) in OB men and 25.1% (ES: 1.63, *large*, *p* < 0.001) in OB women than NOB subjects. In the 2 min step test, obesity significantly impaired performance in OB women (−28.2% vs. NOB women, ES: 0.92, *large*, *p* < 0.001), whereas men showed no significant difference. Additionally, OB men showed a 108.7% (ES: 1.53, *large*, *p* < 0.001) higher handgrip strength than OB women; additionally, NOB men exhibited 28.9% (ES: 0.83, *large*, *p* < 0.001) greater handgrip strength than NOB women. No sex differences were observed in the OB group, conversely, NOB men performed significantly lower repetitions (−17.4%, ES: 0.73, *medium*, *p* = 0.006) in the 30-s chair stand than NOB women. No significant sex differences were observed for the arm curl and 2-min step tests (Table 2). 

The group effect revealed a profound reduction in agility, dynamic balance, and aerobic capacity associated with obesity. The 8-foot up-and-go execution time was significantly higher in the OB group, taking 54.2% (ES: 1.26, *large*, *p* < 0.001) higher for OB men and 104.3% (ES: 1.77, *large*, *p* < 0.001) higher for OB women compared to their NOB peers. Similarly, aerobic endurance (6-minute walk test) was significantly lower: OB men walked 27.5% (ES: 1.58, *large*, *p* < 0.001) less distance, and OB women covered 42.2% (ES: 2.05, *large*, *p* < 0.001) less distance than NOB subjects. 

Upper body flexibility (Back scratch) was also significantly reduced by obesity, decreasing by −76.2% (ES: 1.60, *large*) in OB men and −66.9% (ES: 0.99, *large*) in OB women (relative to NOB, *p* < 0.001). Lower body flexibility (chair sit-and-reach) showed no significant differences between groups.

Regarding sex characteristics, sex-specific differences in mobility emerged exclusively in the obese state: OB men completed the 8-foot up-and-go faster (+22.9%, ES: 0.66, *medium*, *p* < 0.001) and covered more distance (23.4%, ES: 0.64, *medium*, *p* < 0.001) in the 6-minute walk test compared to OB women. No significant sex differences were identified for these parameters within the NOB group, nor for flexibility in either group (Table 2).

PCA for physical capacity reveals that the OB group showed higher fatness scores and marked functional heterogeneity, whereas the NOB group clusters tightly, demonstrating low adiposity paired with higher physical capacity (Figure 1). The suitability of the data for PCA was confirmed by a Kaiser–Meyer–Olkin value of 0.79. Bartlett’s test of sphericity was also statistically significant (χ^2^ = 334.0, df = 36, *p* < 0.001), indicating that the correlation matrix was appropriate for factor extraction. Two components had eigenvalues exceeding 1.0, accounting for 47.1% and 16.5% of the total variance, respectively.

Taken together, the OB group showed broad deficits in strength, agility, and endurance; within that group, women had the greatest mobility and walking endurance limitations, whereas men retained higher absolute handgrip strength. 

### 3.3. Physical Abilities

Obesity significantly compromised global physical performance and independence (Table 2). The PPT score was significantly lower in OB men (−24.3%, ES: 2.74, *large*, *p* < 0.001) and OB women (−25.7%, ES: 2.21, *large*, *p* < 0.001) compared to NOB subjects. IADL score was significantly lower in OB men (−6.2% vs. NOB, ES: 0.59, *medium*, *p*: 0.027), while BADL and the BI scores were significantly lower only in OB women (−3.3% and −4.6%; ES: 0.71, *medium* and ES: 0.71, *medium*; respectively, *p*: 0.006) than NOB. 

Analyzing sex differences within these domains, dimorphism was marginal but statistically present. In the OB group, men maintained slightly higher BADL scores (+1.7%, ES: 0.28, *small*, *p*: 0.042) than women, with no other significant sex discrepancies emerging in performance or independence indices.

OB group shows higher adiposity and highly functional capacity decline, whereas the NOB group clusters tightly, indicating preserved functional autonomy (Figure 2). For the physical ability variables, the Kaiser–Meyer–Olkin index was 0.72, indicating acceptable sampling adequacy. In addition, Bartlett’s test of sphericity reached statistical significance (χ^2^ = 63.6, df = 10, *p* < 0.001), confirming that the correlation matrix was suitable for PCA. Only one component met the eigenvalue-greater-than-one criterion and explained 53.4% of the overall variance. Although the second, third, and fourth components accounted for 22.4%, 12.5%, and 11.8% of the variance, respectively, their eigenvalues remained below 1.0.

Thus, both sexes in the OB group had lower global physical performance, while smaller sex-specific differences in daily autonomy were domain-specific, with basic activities more affected in women and instrumental activities more affected in men.

### 3.4. Physical Activity Level

The PASE score was significantly impaired only in OB women (−36.3% vs. NOB, ES: 1.43, *large*, *p* < 0.001, Table 2). Within the NOB cohort, men exhibited a significantly lower PASE score (−18.8%, ES: 0.77, *medium*, *p* < 0.001) than women.

Figure 3 shows the association between the PCA-derived fatness score and the original PASE score. The OB group shows severe adiposity combined with consistently lower physical activity levels, whereas the NOB cohort exhibits higher but highly variable lifestyle activity (Figure 3). Regarding the fatness index, the adequacy of the dataset for PCA was supported by a Kaiser–Meyer–Olkin value of 0.64 and a significant Bartlett’s test of sphericity (χ^2^ = 553.1, df = 6, *p* < 0.001). The analysis retained a single component with an eigenvalue above 1.0, which accounted for 77.5% of the total variance. The second component explained an additional 17.9% of the variance, although its eigenvalue was lower than 1.0.

Overall, reduced self-reported activity was most apparent in women in the OB group.

### 3.5. Quality of Life

The SF-12 quality of life score was significantly reduced only in OB men (−10.8% vs. NOB, ES: 1.07, *large*, *p* < 0.001, Table 2). In addition, NOB men showed a higher SF-12 quality of life score (+7.9%, ES: 0.77, *medium*, *p* < 0.001) than NOB women. 

Figure 4 shows the association between the PCA-derived fatness score and the original SF-12 score. The severe adiposity in the OB group consistently constrains maximum health perception scores; in contrast, health-related quality of life (SF-12) remains highly variable within the NOB cohort. The fatness score used in this analysis was the same PC1 score used in Figure 3; therefore, the KMO statistic, Bartlett’s test, eigenvalues, and proportion of explained variance refer to the same fatness PCA.

Overall, the quality-of-life findings were domain-specific rather than a uniform reduction in perceived health.

### 3.6. Sarcopenic Obesity Status

Using the study-specific operational application of the ESPEN/EASO framework described in Section 2.7, all participants in the obese cohort (both men and women) were classified as presenting an SO phenotype. This classification reflects the operational decision rules adopted in the present study and should not be interpreted as definitive confirmation of the complete consensus diagnostic pathway.

## 4. Discussion

Within the study-specific operational application of the ESPEN/EASO framework, all older adults with obesity included in the present study were classified as presenting an SO phenotype. This finding should not be interpreted as definitive clinical confirmation according to the complete ESPEN/EASO diagnostic algorithm, but rather as an operational classification based on the available measures. Nevertheless, the observation supports the view that, in geriatric obesity, excess adiposity should not be interpreted as an isolated metabolic condition but as part of a broader phenotype that includes reduced muscle function, compromised mobility and early loss of autonomy [6,7,32].

A second key finding is the clear sex-specific divergence in adiposity distribution and functional consequences. OB men had a higher absolute and relative VAT burden and a higher ABSI-RR than OB women. This suggests that, at comparable BMI levels, older obese men may accumulate a more adverse abdominal visceral phenotype. These results are consistent with evidence that BMI alone is insufficient to characterize cardiometabolic risk and that body shape indices, including BRI and ABSI, provide complementary information on central adiposity and mortality risk [11,12,13].

In contrast, OB women showed more severe functional impairment despite having lower VAT mass. Their lower performance in the 8-foot up-and-go and 6-minute walk test suggests that mobility and endurance may be particularly vulnerable in older obese women. This finding may reflect a lower baseline absolute FFM, sex-related difference in muscle quality, and a lower functional reserve before the threshold for disability is reached [33,34]. Fat infiltration into skeletal muscle and reduced muscle quality have been linked to poorer physical performance in older women with obesity [8,35].

The autonomy outcomes further reinforce this interpretation. Although PPT scores were reduced in both OB men and women, impairments in BADL and the Barthel index were evident mainly in OB women, whereas OB men showed impairment primarily in IADL. This pattern may indicate greater vulnerability of women to limitations in basic self-care, while functional impairment in men may initially be more apparent in complex activities required for independent community living. This interpretation is consistent with previous evidence linking obesity to functional decline and disability in older adults, with particularly pronounced effects among women [36,37]. Clinically, these findings argue against a uniform rehabilitation prescription for all older adults with obesity and support interventions tailored to sex-specific functional profiles.

The apparent discrepancy between objective impairment and perceived health status is also noteworthy. OB women showed poorer mobility and basic autonomy, whereas SF-12 scores were significantly reduced only in OB men. This mismatch may reflect sex-related differences in the appraisal of health and functional decline, including different psychological responses, expectations regarding independence, and thresholds for perceiving and reporting health loss [38,39]. Because the SF-12 captures perceived health rather than objective function, sex-specific coping strategies and social roles should be considered when interpreting quality-of-life outcomes [40].

The PCA plots provide a useful multidimensional synthesis of these findings. NOB participants clustered toward lower fatness and better functional status, whereas OB participants were characterized by greater fatness and marked heterogeneity in physical function. This variability is clinically relevant, as it indicates that older adults with similarly severe adiposity may exhibit substantially different degrees of functional impairment. Accordingly [6,41], rehabilitation planning should incorporate direct assessments of muscle strength and physical performance rather than relying on BMI alone.

These results have practical implications. For older women with SO, intervention should prioritize lower-limb resistance training, gait and balance training, progressive mobility exposure and strategies to maintain BADL independence. For older men, programs should include visceral fat reduction through nutrition and aerobic/resistance exercise, while also addressing perceived health, motivation, and psychological adaptation to functional decline. In both sexes, weight loss interventions should preserve or improve muscle mass and strength, because weight loss without adequate exercise and protein support may exacerbate sarcopenia [42,43].

Some limitations should be acknowledged. First, the cross-sectional design prevents causal inference. Second, the obese sample size came from a clinical rehabilitation context, limiting generalizability to community-dwelling older adults. Third, body composition was assessed using BIA rather than DXA; therefore, estimates of lean mass and VAT should be interpreted with caution. The SO classification was an operational application of the ESPEN/EASO framework based on BIA-derived body composition and should therefore be interpreted accordingly. Finally, the age difference between OB women and NOB women may have contributed to some functional differences.

## 5. Conclusions

In the present clinical sample, all participants with obesity were classified as presenting an SO phenotype according to the study-specific operational application of the ESPEN/EASO framework and exhibited distinct sex-specific adiposity and functional profiles. Men displayed a higher geometric visceral adiposity risk profile and lower perceived health-related quality of life, whereas women displayed greater mobility impairment and earlier compromise of basic autonomy. These findings highlight the need for sex-differentiated, multidisciplinary interventions that combine adiposity reduction with preservation or restoration of muscle strength, mobility and functional independence.

## Figures and Tables

**Table 1:** Anthropometric characteristics and body composition of subjects.

	Obese		Non-Obese			*p*	
	Men (n:27)	Women (n:18)	Men (n:18)	Women (n:22)	Group	Sex	Group × Sex
Age (y)	71.8 ± 4.9	73.2 ± 4.0 ^#^	70.8 ± 4.4	67.4 ± 2.1 °	<0.001	0.268	0.010
Body mass (kg)	113.4 ± 18.5 *^§^	94.0 ± 15.6 ^#^	70.6 ± 8.0	60.6 ± 7.8 °	<0.001	<0.001	0.149
Height (m)	1.72 ± 0.07	1.55 ± 0.07	1.76 ± 0.08 *	1.64 ± 0.09 *	0.370	<0.001	0.370
BMI (kg m^−2^)	38.1 ± 5.5 ^§^	39.2 ± 5.6 ^#^	22.8 ± 1.2	22.5 ± 1.3	<0.001	0.681	0.467
WC (m)	1.27 ± 0.13 ^§^	1.14 ± 0.09 *^#^	0.85 ± 0.06	0.82 ± 0.05	<0.001	<0.001	0.041
WHtR	0.74 ± 0.07 ^§^	0.74 ± 0.07 ^#^	0.49 ± 0.03	0.5 ± 0.04	<0.001	0.607	0.676
BMFI	2104 ± 755 ^§^	2434 ± 765 ^#^	443 ± 78	574 ± 127	<0.001	0.088	0.460
BRI	9.1 ± 2.2 ^§^	9.1 ± 1.9 ^#^	3.1 ± 0.5	3.4 ± 0.7	<0.001	0.738	0.752
VAT mass (kg)	6.01 ± 3.3 *^§^	2.91 ± 1.03 ^#^	0.39 ± 0.21	0.58 ± 0.13	<0.001	0.003	0.001
VAT mass (%)	11.2 ± 3.4 *^§^	5.9 ± 1.6 ^#^	2.8 ± 1.9	3.1 ± 0.5	<0.001	<0.001	<0.001
ABSI	0.09 ± 0.01 *^§^	0.08 ± 0.01	0.08 ± 0.01	0.08 ± 0.01	0.019	0.017	0.016
ABSI-RR	1.10 ± 0.34 *	0.86 ± 0.23	0.87 ± 0.25	0.98 ± 0.29	0.418	0.360	0.012
FFM (kg)	63.7 ± 7.7 *^§^	44.3 ± 4.0	54.6 ± 8.5	42.0 ± 8.0 °	<0.001	<0.001	0.065
FFM (%)	57.3 ± 6.2 *^§^	47.6 ± 5.9 ^#^	77.7 ± 4.8	68.9 ± 5.3 °	<0.001	<0.001	0.698
FM (kg)	48.8 ± 13.4 ^§^	50.4 ± 13.6 ^#^	15.9 ± 2.7	18.6 ± 2.9	<0.001	0.365	0.819
FM (%)	42.7 ± 6.2 *^§^	52.5 ± 5.9 ^#^	22.3 ± 4.4	31.1 ± 5.3 °	<0.001	<0.001	0.543

Data are presented as mean ± standard deviation; BMI: body mass index; WC: waist circumference; WHtR: waist-to-height ratio; BMFI: body mass fat index; BRI: body roundness index; VAT: visceral adipose tissue; ABSI: A body shape index; ABSI-RR: A body shape index relative risk; FFM: fat-free mass; FM: fat mass. § difference between OB men vs. NOB men; # difference between OB women vs. NOB women; * difference between OB men vs. OB women; ° difference between NOB men vs. NOB women.

**Table 2:** Physical capacities, physical abilities, physical activity level and quality of life of subjects.

	Obese		Non-Obese			*p*	
	Men (n:27)	Women (n:18)	Men (n:18)	Women (n:22)	Group	Sex	Group × Sex
Right-hand grip strength (kg)	26.5 ± 10.1 *^§^	12.7 ± 7.8 ^#^	36.6 ± 10.9	28.4 ± 8.6 °	<0.001	<0.001	0.206
Left-hand grip strength (kg)	24.6 ± 8.8 *^§^	10.6 ± 7.9 ^#^	35.5 ± 10.5	26.7 ± 9.8 °	<0.001	<0.001	0.222
*Senior Fitness Test*							
30-Second Chair Stand (reps)	10.8 ± 2.6 ^§^	10.2 ± 3.0 ^#^	15.7 ± 5.1	19.0 ± 3.8 °	<0.001	0.130	0.021
Arm Curl (reps)	14.2 ± 4.6 ^§^	14.9 ± 2.7 ^#^	18.1 ± 4.1	19.9 ± 3.4	<0.001	0.151	0.550
2-Min step (reps)	79.6 ± 31.5	66.2 ± 36.6 ^#^	85.6 ± 15.4	92.2 ± 16.0	0.012	0.590	0.111
Chair Sit-and-Reach (cm)	−0.9 ± 12.4	2.0 ± 9.4	1.6 ± 11.8	4.6 ± 8.1	0.405	0.327	0.980
Back Scratch (cm)	−21.5 ± 11.3 ^§^	−16.7 ± 11.5 ^#^	−5.3 ± 8.8	−5.6 ± 10.9	<0.001	0.397	0.315
8-Foot Up-and-Go (s)	7.4 ± 2.8 *^§^	9.6 ± 3.8 ^#^	4.8 ± 0.8	4.7 ± 0.9	<0.001	0.058	0.039
6-Min Walk (m)	406 ± 113 *^§^	329 ± 129 ^#^	560 ± 78	569 ± 104	<0.001	0.167	0.081
PPT	21.2 ± 3.5 ^§^	20.8 ± 4.6 ^#^	28 ± 0	28 ± 0	<0.001	0.726	0.726
BADL	5.9 ± 0.3 *	5.8 ± 0.4 ^#^	6 ± 0	6 ± 0	0.006	0.109	0.109
IADL	7.5 ± 1.2 ^§^	7.6 ± 0.8	8 ± 0	8 ± 0	0.021	0.817	0.817
BI	97.7 ± 5.1	95.4 ± 5.9 ^#^	100 ± 0	100 ± 0	<0.001	0.190	0.190
PASE	143.5 ± 29.9	130.7 ± 35.0 ^#^	166.5 ± 30.2	205.1 ± 64.6 °	<0.001	0.186	0.009
SF-12	30.5 ± 2.8 ^§^	30.5 ± 3.5	34.2 ± 4.0	31.7 ± 2.3 °	0.001	0.087	0.077

Data are presented as mean ± standard deviation; PPT: physical performance test; BADL: basic activities of daily living; IADL: instrumental activities of daily living; BI: Barthel index; PASE: physical activity scale for the elderly; SF-12: 12-item short-form health survey; § difference between OB men vs. NOB men; # difference between OB women vs. NOB women; * difference between OB men vs. OB women; ° difference between NOB men vs. NOB women.

## Data Availability

The raw data will be deposited in Zenodo after manuscript acceptance and will be available from the corresponding authors upon reasonable request.

## Abbreviations

The following abbreviations are used in this manuscript:

ABSI	A body shape index
ABSI-RR	A body shape index-associated relative risk
BADL	basic activities of daily living
BIA	bioelectrical impedance analysis
BI	Barthel index
BMI	body mass index
BMFI	body mass fat index
BRI	body roundness index
DXA	dual-energy X-ray absorptiometry
EASO	European Association for the Study of Obesity
ESPEN	European Society for Clinical Nutrition and Metabolism
FFM	fat-free mass
FM	fat mass
HGS	handgrip strength
IADL	instrumental activities of daily living
NOB	non-obese
OB	obese
PASE	physical activity scale for the elderly
PCA	principal component analysis
PPT	physical performance test
SF-12	12-item short-form health survey
SFT	senior fitness test
SO	sarcopenic obesity
VAT	visceral adipose tissue
WC	waist circumference
WHtR	waist-to-height ratio
